# Identifying Unique Subgroups of Emotional and Behavioral Presentations in a Large Inpatient and Community Sample of Autistic Youth

**DOI:** 10.1002/aur.70307

**Published:** 2026-06-26

**Authors:** Safaa Eldeeb, Jessie Northrup, Caitlin Conner, Shalini Sivathasan, Ligia Antezana, Matthew Siegel, Carla Mazefsky

**Affiliations:** ^1^ Department of Psychiatry University of Pittsburgh Pittsburgh Pennsylvania USA; ^2^ Boston College Boston Massachusetts USA; ^3^ Boston Children's Hospital, Franciscan Children's Hospital Tufts University School of Medicine Boston Massachusetts USA

**Keywords:** aggression, autism, behavioral phenotyping, emotion dysregulation inventory, machine learning, random forests ensemble clustering

## Abstract

Autistic youth exhibit wide variability in emotional and behavioral challenges, yet few studies have identified meaningful subgroups based on these profiles. This study applied a random forests ensemble clustering algorithm to item‐level parent‐report data from the Emotion Dysregulation Inventory (EDI) and the Child Behavior Checklist (CBCL) in a combined sample of 1311 autistic youth (ages 6–17), drawn from the Autism Inpatient Collection (*n* = 446) and the Interactive Autism Network (*n* = 865). Four distinct subgroups emerged: Global High (GH; 22%), characterized by elevated scores across all four subscales (EDI‐Reactivity, EDI‐Dysphoria, CBCL Internalizing, and CBCL Externalizing); High‐Reactivity Dominant (H‐RD; 41%), marked primarily by high emotional reactivity; Moderate‐Internalizing Dominant (M‐ID; 18%), with elevated internalizing scores; and Global Low (GL; 19%), showing uniformly low scores. Feature importance analyses identified EDI‐Reactivity and CBCL Aggression items as the strongest drivers of subgroup membership. No significant differences were found across subgroups in age, sex, race, or ethnicity. However, subgroups with greater emotional and behavioral challenges were associated with lower household income, single‐parent status, and higher rates of family psychiatric history. These findings suggest that emotional reactivity and aggression severity are key differentiating features among autistic youth, and that sociodemographic and family mental health factors meaningfully shape emotional and behavioral outcomes.

## 1 Introduction

### 1.1 Emotional and Behavioral Presentations in Autistic People

Autistic people exhibit a diverse array of emotional and behavioral challenges, which differ significantly in terms of their prevalence and severity (Lai et al. 2019; Levy et al. 2010; Bauminger et al. 2010; Simonoff et al. 2008; Salazar et al. 2015; Lai 2023). These emotional and behavioral presentations can have a significant impact on the individual's quality of life and can also affect their ability to participate in daily activities and social interactions. Many autistic individuals enter treatment, not primarily for autistic traits, but due to these emotional and behavioral challenges (Farley et al. 2009; Joshi et al. 2010). Emotional and behavioral concerns drive the need for intensive services such as psychiatric hospitalizations and are also associated with serious consequences such as suicidality (Conner et al. 2021; Conner, Golt, et al. 2020).

Emotional and behavioral concerns are often broken down into internalizing problems and externalizing problems. Internalizing problems involve emotional distress and withdrawal and may include symptoms such as anxiety, depression, social withdrawal, and low self‐esteem. Externalizing problems are outwardly directed and typically observable by others, including behaviors such as aggression, oppositionality, and impulsivity (Bauminger et al. 2010; Salazar et al. 2015) Emotional and behavioral concerns have also been commonly conceptualized in autism in terms of the degree of emotion dysregulation (ED). ED is a transdiagnostic feature that has commonly been measured in autism in terms of reactivity (rapidly escalating, intense, poorly regulated negative emotional reactions such as outbursts) and dysphoria (poor upregulation of positive affect, unease, and sadness) (Mazefsky et al. 2013; Cai et al. 2018; Cibralic et al. 2019; Sivathasan et al. 2024).

Internalizing problems are particularly prevalent among autistic individuals. A meta‐analysis of 96 studies estimated pooled prevalence rates of 20% for anxiety disorders and 11% for depression. Similarly, externalizing problems are common, with pooled prevalence rates of 28% for attention‐deficit/hyperactivity disorder (ADHD) and 12% for disruptive behavior disorders (Bauminger et al. 2010; Salazar et al. 2015). ED also appears markedly elevated in autism. In one study, community and inpatient samples of autistic individuals showed rates of 53.2% and 92.9%, respectively, exceeding clinical cutoffs for emotional reactivity compared to the general US rate of 13.6% (Conner et al. 2021). Likewise, 32.1% of the community autistic group and 71.3% of the inpatient autistic group scored in the clinical range for dysphoria, compared with 15.1% in the general sample (Conner et al. 2021).

Taken together, internalizing problems, externalizing problems, and ED are all reported at high rates in autistic samples. Nonetheless, these problems are clearly not universal within autism. Further, many autistic individuals exhibit a combination of these challenges. Identification of meaningful subgroups of autistic individuals with similar emotional and behavioral profiles would provide opportunities to identify risk and protective factors and develop more tailored prevention and intervention supports.

### 1.2 Previous Studies on Subgrouping of Autistic Individuals

Prior research has demonstrated the potential of subgrouping approaches to identify meaningful subgroups within the autism spectrum, focusing on diagnostic autism characteristics, developmental characteristics, and medical profiles (Bitsika et al. 2008; Hu and Steinberg 2009; Sacco et al. 2012; Zheng et al. 2019). Most studies have focused on clustering autistic individuals based on genetic or medical data (Cuccaro et al. 2012; Doshi‐Velez et al. 2014; Wiggins et al. 2011), sensory difficulties (Uljarević et al. 2016; Ben‐Sasson et al. 2008; Tomchek et al. 2018; Ausderau et al. 2016; Kirby et al. 2022) and co‐occurring medical conditions. However, there has been a notable lack of studies that focus on subgrouping autistic individuals based on their emotional and behavioral profiles, despite the significant impact these factors can have on both family dynamics and individual well‐being. This gap is significant, as many individuals experience substantial mental health issues which can often take precedence over core autistic traits (Farley et al. 2009; Joshi et al. 2010).

Two studies included behavioral variables in subgroups. Stevens et al. identified seven distinct subgroups based on behavioral variables (aggression, self‐injury, disruption, elopement, stereotypy, tantrums, non‐compliance, and obsession), indicating that while multiple behaviors were often present, a dominant behavior typically emerged in most cases (Stevens et al. 2017). Another study included two broadband measures of behavioral and emotional characteristics, measures of autism characteristics, adaptive behavior, and parental autism characteristics (Matta et al. 2018). Analysis identified three subgroups representing low, medium, and high symptom severity, though this was across measures and did not suggest specificity in patterns related to emotional and behavioral characteristics (Matta et al. 2018).

This study had three primary objectives: (1) to identify distinct subgroups of autistic youth sharing similar emotional and behavioral profiles using machine learning cluster analysis; (2) to determine which specific clinical features most strongly drove subgroup membership; and (3) to examine whether subgroups differed on sociodemographic and clinical characteristics.

## 2 Methods

All study procedures were approved by the University of Pittsburgh Institutional Review Board (Protocol #19050171) (Mazefsky, Day, et al. 2018) and the IRBs of each institution that contributed to the Autism Inpatient Collection (Siegel et al. 2015), and all participants provided their consent to participate in accordance with the declaration of Helsinki.

### 2.1 Participants

This study utilized two data sets to capture the full range of emotional and behavioral concerns in autism. The first dataset was collected through the Interactive Autism Network (IAN), an online registry in the United States for individuals with professionally diagnosed autism. For this study, participants were selected based on a Social Communication Score‐Lifetime Version (SCQ) (Rutter et al. 2003) higher than 12 and age range of 6–17 years old, yielding 1176 individuals. IAN data were collected as part of an ongoing registry; the data used in this study reflect a cross‐sectional assessment wave conducted in 2016.

The second dataset was the Autism Inpatient Collection (AIC), a six‐site study of autistic youth (ages 4–20) admitted to specialized inpatient psychiatric units. The complete methodology for the AIC has been published (Siegel et al. 2015). Eligibility required an SCQ score above 12 or showing a high suspicion of autism as determined by the inpatient clinical treatment team. Inclusion in the AIC dataset also required confirmation of autism diagnosis through the research‐reliable administration of the Autism Diagnostic Observation Schedule‐2 (Lord et al. 2000), and a clinical diagnosis of autism based on DSM‐5 criteria. Exclusion criteria included the unavailability of an English‐proficient caregiver or status as a prisoner for the autistic individual. AIC data collection spanned multiple phases beginning in 2014. The CBCL was introduced in Phase 2 of data collection (October 2015); accordingly, the data used in the present analyses were drawn from Phases 2 and 3, covering the period October 2015 through September 2022. Although the full sample included 1356 individuals, only 446 had complete Child Behavior Checklist data due to its later introduction.

Participants with incomplete item‐level EDI or CBCL data were excluded from analyses to ensure reliable cluster solutions, as missing values can distort distance calculations and destabilize cluster structures (Mazza et al. 2015). In the IAN sample, exclusions were primarily due to missing CBCL item‐level responses. In the AIC sample, missingness was predominantly structural: the CBCL was introduced beginning in Phase 2 of data collection, and consequently Phase 1 participants lacked CBCL data entirely. For this study, we included only participants aged 6–17 years old with complete item‐level data (865 from IAN; 446 from AIC), ensuring reliable cluster analyses. Missing data would hinder our ability to identify meaningful patterns and subgroups, making it essential to exclude these participants from the analysis. Therefore, our final sample consisted of 1311 participants, with an average age of 12.35 years (SD = 3.3 years). The sociodemographic and clinical characteristics of the study sample are presented in Table 1. Data is available upon reasonable request to the corresponding author.

**TABLE 1:** Sociodemographic and clinical characteristics of the study sample.

Age in years, mean (SD), *n* = 1198	12.3 (3.3)
Sex assigned at birth, *n* (%), *n* = 1311	
Male	1072 (81.8%)
Female	239 (18.2%)
Race*, *n* (%), *n* = 1307	
Asian	28 (2.1%)
Black	95 (7.3%)
Native American	18 (1.4%)
Native Hawaiian	2 (0.15%)
White	1120 (85.7%)
Another race	38 (3%)
Ethnicity, *n* (%), *n* = 1301	
Hispanic/Latino, *n* = 104	104 (8%)
Non‐Hispanic/Non‐Latino, *n* = 1197	1197 (92%)
Verbal ability, *n* (%), *n* = 1305	
Nonverbal	703 (53.8%)
Verbal	602 (46.2%)
SCQ, mean (SD), *n* = 1229	22.84 (6.5)
Intellectual disability, *n* = 1171	605 (51.6%)
Household income, *n* (%), *n* = 1213	
Less than $20,000	132 (10.9%)
$20,000–$50,000	301 (24.8%)
$51,000–$80,000	275 (22.7%)
$81,000–$100,000	132 (10.9%)
Over 100,000	373 (30.8%)
Parental marital status, *n* (%), *n* = 1288	
Single	89 (7%)
Married	946 (73%)
Divorced	161 (12.5%)
Separated	31 (2.4%)
Widowed	22 (1.7%)
Domestic partner	39 (3%)
CBCL Internalizing problems T‐score, mean (SD), *n* = 1311	62.4 (9.6)
CBCL Externalizing problems T‐score, mean (SD), *n* = 1311	56.9 (7.3)
EDI Reactivity T‐score, mean (SD), *n* = 1311	63.75 (10.7)
EDI Dysphoria T‐score, mean (SD), *n* = 1311	54.3 (9)

*participants were able to “check all that apply” for US Census race categories, thus values do not total to 100%. We have 6 participants who checked more than one category: two of them (Asian and Native American), another two (Native American and Native Hawaiian), one participant with (Asian and Black), and finally one participant with (Black and Native Hawaiian).

### 2.2 Measures

Caregivers reported child and caregiver sociodemographic characteristics (child age, sex assigned at birth, racial and ethnic identity; caregiver marital status, household income) and immediate family psychiatric history (bipolar disorder, depression, anxiety, ADHD, PTSD). Clinical features included verbal ability (ADOS‐2 module for inpatient participants; caregiver report for community participants), intellectual ability (Leiter‐3 NVIQ for inpatients; caregiver‐reported IQ ranges for community participants), and autism‐related characteristics (Social Communication Questionnaire‐Lifetime) (Rutter et al. 2003). Emotion dysregulation was assessed using the Emotion Dysregulation Inventory (EDI; Reactivity and Dysphoria subscales) (Mazefsky, Yu, et al. 2018), and psychiatric symptoms were measured using caregiver‐reported Child Behavior Checklist (CBCL) (Achenbach and Rescorla 2001) internalizing and externalizing items. Additional instrument details, scoring procedures, psychometric properties, and rationale for analytic use are provided in Supporting Information S1.

### 2.3 Data Analytic Plan

#### 2.3.1 Aim 1: Clustering Analysis

To identify subgroups of autistic youth who share similar emotional and behavioral presentations, we applied an ensemble clustering approach using a random forest–based machine learning method to item‐level data from the CBCL externalizing and internalizing scales and the EDI reactivity and dysphoria subscales (i.e., individual survey items) (Alhusain and Hafez 2017; Salman et al. 2024). A cluster ensemble combines the results of multiple independent clustering runs—called base clusterings—into a single, stable consensus solution, reducing sensitivity to random initialization and improving the reproducibility of results. Cluster ensemble is used to enhance the effectiveness and stability of the clustering algorithm. The technique comprises two main elements: an ensemble constructor, which generates several data partitions, known as “base clustering” and a consensus function, which merges the base clustering to produce reliable clustering (Alhusain and Hafez 2017). This method is known to improve the clustering outcomes compared to a single clustering approach. Random forests clustering is known to be the most robust clustering method when dealing with categorical data (Salman et al. 2024; Breiman 2001). Random forests utilize multiple decision trees; each trained on a different random subset of the data and features to improve model performance and prevent overfitting. The final prediction is obtained by combining the predictions of all the trees in the forest. Random forests clustering is also robust to multicollinearity among input features, because each tree selects a random subset of features at each split, reducing the influence of any single highly correlated variable pair. By leveraging the diversity of the decision trees, random forests can improve the accuracy and robustness of the model.

Analysis of Variance (ANOVA) was carried out to identify significant differences between the resulting subgroups in emotional and behavioral presentations. Although we used the survey items for the clustering algorithm, we analyzed the score‐level data to interpret the subgroups more meaningfully. Specifically, we analyzed subgroup differences in CBCL externalizing problems, CBCL internalizing problems, as well as the EDI‐reactivity and dysphoria subscales. Significant results were followed up with post hoc pairwise comparisons with Tukey's HSD correction for multiple comparisons to identify specific subgroup pairs that exhibited significant differences.

#### 2.3.2 Aim 2: Key Clinical Features for Clustering Membership

Silhouette scores—a metric ranging from −1 to +1 that measures how similar each observation is to its own cluster relative to other clusters, with higher scores indicating better‐defined separation—were utilized to determine the optimal number of subgroups. Additionally, a mean decrease accuracy (MDA) feature selection algorithm was used to identify the clinical features (i.e., survey items) that had the strongest influence on the clustering decisions. The MDA algorithm assesses the decrease in accuracy when comparing the original clustering results to results obtained after randomly permuting a clinical feature. Clinical features that exhibit a higher decrease in accuracy are considered more significant in the clustering results (Babu and Li 2016).

#### 2.3.3 Aim 3: Sociodemographic and Clinical Features Group Differences

We utilized Chi‐square tests of independence for categorical variables and ANOVAs for continuous variables to investigate subgroup differences in the sociodemographic variables and clinical features presented in Table 1, in addition to family psychiatric history. Significant results were followed up with pairwise comparisons utilizing either ANOVA with Tukey's HSD or Chi‐square tests with Bonferroni correction to identify specific subgroup pairs that exhibited significant differences.

### 2.4 Sensitivity Analyses

To address potential concerns about the impact of missing data on the stability of our findings, we conducted a supplemental sensitivity analysis using multiple imputation by chained equations (van Buuren and Groothuis‐Oudshoorn 2011), generating 20 imputed datasets across the full available sample. The random forests clustering was re‐run on each imputed dataset, and the resulting subgroup solutions were compared with our primary complete‐case analysis. Results of this sensitivity analysis are reported in the Supporting Information (Supporting Information S2).

## 3 Results

### 3.1 Aim 1: Clustering Analysis

**FIGURE 1:**
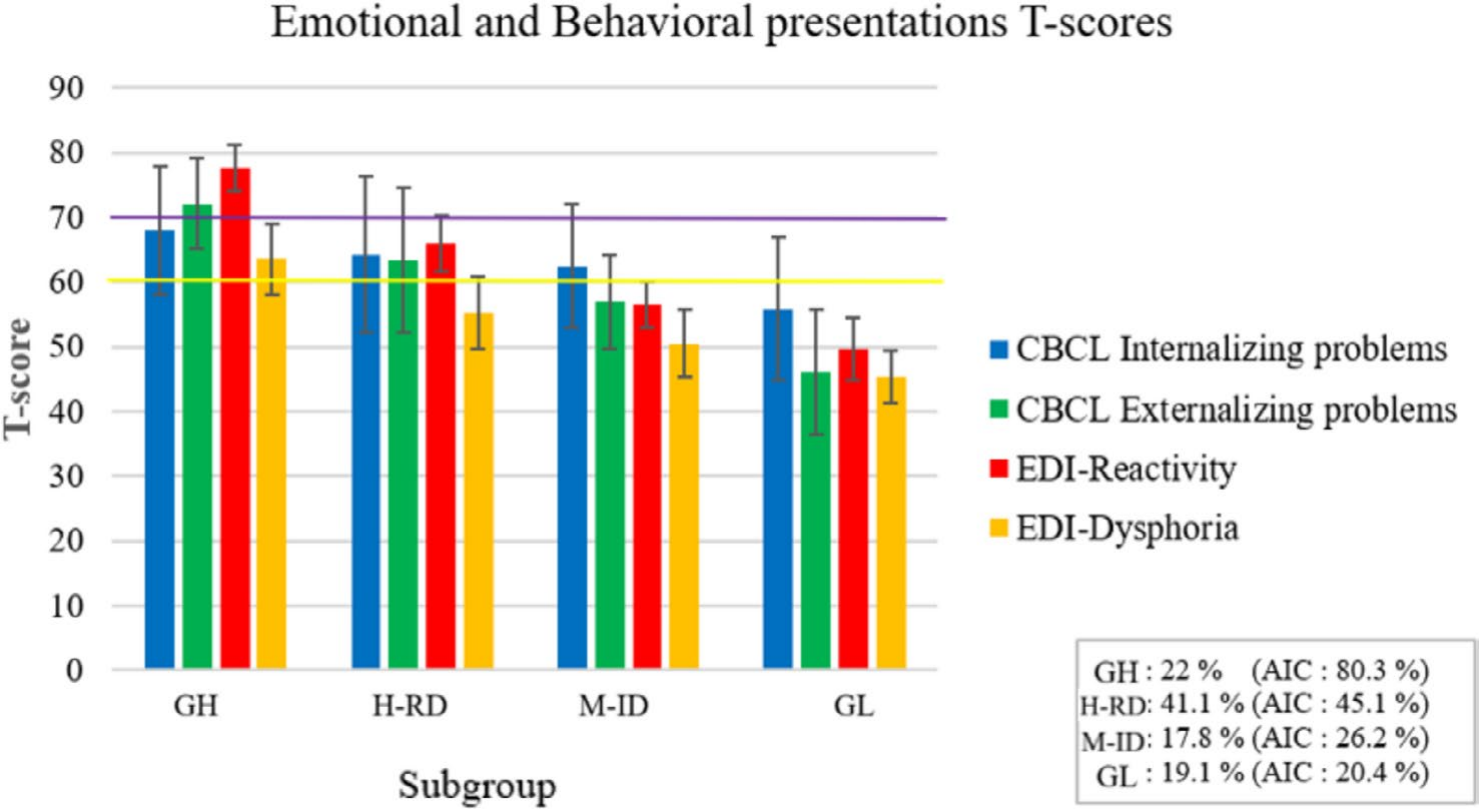
T‐Score mean and standard deviation of the child behavior checklist—internalizing and externalizing problems subscales and emotion dysregulation inventory–reactivity and dysphoria for machine learning derived autistic subgroups. The horizontal yellow line shows the threshold to the clinical cut off scores of the EDI, while the purple line shows the threshold to the clinical cut off score of the CBCL internalizing and externalizing problems.

The random forests machine learning clustering analysis revealed that the four‐subgroup solution was the best fit, indicating the presence of four distinct groups of autistic children and adolescents based on item‐level CBCL and EDI data (see Figure 1). The first subgroup, referred to as Global High (GH), demonstrated elevated scores across all four CBCL and EDI subscales. The second subgroup, referred to as High‐Reactivity Dominant (H‐RD), displays elevated scores, particularly for the Reactivity subscale, which emerges as the most prominent feature of this group. The third subgroup, labeled Moderate‐Internalizing Dominant (M‐ID), exhibits moderate scores across the four subscales, with the Internalizing subscale as most elevated. Finally, the fourth subgroup, termed Global Low (GL), is characterized by low scores across all subscales.

Differences between the four subgroups in the total subscales' scores were assessed using ANOVA tests, which demonstrated significant differences in internalizing problems (*F* (3, 1307) = 371.4), externalizing problems (*F* (3, 1307) = 1384), EDI‐Reactivity (*F* (3, 1307) = 782.7), and EDI‐Dysphoria (*F* (3, 1307) = 739), across the four subgroups, all with *p*s < 0.0001, see Table 2. Furthermore, post hoc pairwise comparisons with Tukey's HSD correction demonstrated that all four subgroups exhibited significant differences from one another across all four subscales. The observed pairwise differences suggest a consistent hierarchy across the four subscales, with GH exhibiting greater scores than H‐RD, which in turn exceed those of M‐ID, followed by GL, thereby establishing a clear gradient of severity.

**TABLE 2:** Clinical characteristics of autistic participants by subgroup.

	GH	H‐RD	M‐ID	GL	*X* ^2^/*F*	*p*
	*N* = 289	*N* = 539	*N* = 233	*N* = 250		
Age in years, mean (SD), *n* = 1198	12.4 (3.5)	13 (3.6)	12.7 (3.7)	13.1 (3.6)	0.02	0.99
Sex assigned at birth, *n* (%), *n* = 1311						
Male, *n* = 1072	234 (81.6%)	432 (79.9%)	195 (85.4%)	211 (82.4%)	3.31	0.34
Female, *n* = 239	55 (18.4%)	107 (20.1%)	38 (14.6%)	39 (17.6%)		
Ethnicity, *n* (%), *n* = 1301						
Hispanic/Latino, *n* = 104	21 (7.4%)	48 (9%)	17 (6.4%)	18 (7.2%)	9.02	0.06
Non‐Hispanic/Non‐Latino, *n* = 1197	262 (92.6%)	487 (91%)	216 (93.6%)	232 (92.8%)		
Race, *n* (%), *n* = 1309						
White, *n* = 1151	262 (87.9%)	475 (83%)	203 (78%)	211 (84%)	8.12	0.23
CBCL Internalizing problems T‐score, mean (SD), *n* = 1311	67.9 (10)	64.2 (12.2)	62.4 (9.6)	55.79 (11)	371.4	**< 0.0001**
CBCL externalizing problems T‐score, mean (SD), *n* = 1311	72 (6.9)	63.3 (11)	56.9 (7.3)	46.2 (9.6)	1384	**< 0.0001**
EDI reactivity T‐score, mean (SD), *n* = 1311	77.6 (3.4)	65.9 (4.3)	56.6 (3.5)	49.6 (4.8)	782.7	**< 0.0001**
EDI dysphoria T‐score, mean (SD), *n* = 1311	63.5 (5.46)	55.2 (5.56)	50.5 (5.1)	45.36 (4)	739	**< 0.0001**
Above CBCL Internalizing problems clinical cut‐off, *n* (%)	200 (69%)	318 (59%)	115 (49%)	5 (0.2%)		
Above CBCL externalizing problems clinical cut‐off, *n* (%)	263 (91%)	283 (52.5%)	23 (1%)	0		
Above EDI reactivity clinical cut‐off, *n* (%)	289 (100%)	487 (90.35%)	48 (20.6%)	2 (0.8%)		
Above EDI dysphoria clinical cut‐off, *n* (%)	192 (66%)	108 (20%)	14 (6%)	1 (0.4%)		
Clinical features						
Verbal ability, *n* (%), *n* = 1305						
Non/minimally verbal	136 (47.2%)	285 (52.9%)	136 (58.6%)	146 (59%)	10.13	0.0175
Verbal	152 (52.8%)	253 (47.1%)	96 (41.4%)	101 (41%)		
Intellectual disability, *n* (%), *n* = 1171	109 (39%)	260 (48%)	104 (46%)	132 (51%)	5.6	0.17
SCQ, mean (SD), *n* = 1229	24.6 (6.2)	22.7 (6.9)	22.4 (6.7)	21.6 (6.4)	0.74	0.5
Family psychiatric history						
Bipolar, *n* (%), *n* = 1273						
No	183 (67.3%)	410 (78.5%)	189 (82.2%)	217 (87.1%)	33.13	**< 0.0001**
Yes	89 (32.7%)	112 (21.5%)	41 (17.8%)	32 (12.9%)		
Depression, *n* (%), *n* = 1270						
No	89 (33%)	190 (36.5%)	90 (39.1%)	122 (49%)	15.98	0.**0011**
Yes	181 (67%)	331 (63.5%)	140 (60.9%)	127 (51%)		
Anxiety, *n* (%), *n* = 1271						
No	93 (34%)	164 (31.6%)	86 (37.2%)	103 (41.4%)	7.64	0.054
Yes	179 (66%)	355 (68.4%)	145 (62.8%)	146 (58.6%)		
PTSD, *n* (%), *n* = 1266						
No	193 (72%)	396 (76.3%)	182 (79.1%)	213 (85.5%)	14.70	0.**002**
Yes	75 (28%)	123 (23.7%)	48 (20.9%)	36 (14.5%)		
ADHD, *n* (%), *n* = 1267						
No	140 (52.4%)	267 (51.2%)	133 (57.8%)	173 (69.5%)	24.81	**< 0.0001**
Yes	127 (47.5%)	254 (48.8%)	97 (42.2%)	76 (30.5%)		
Household income, *n* (%), *n* = 1213						
Less than $20,000	42 (15.7%)	60 (12%)	15 (7.1%)	15 (6.4%)	50.69	**< 0.0001**
$20,000 to $50,000	69 (25.7%)	141 (28.3%)	47 (22.2%)	44 (18.7%)		
$51,000 to 80,000	60 (22.4%)	107 (21.4%)	57 (26.9%)	51 (21.7%)		
$81,000 to 100,000	33 (12.3%)	53 (10.64%)	26 (12.3%)	20 (8.5%)		
Over 100,000	64 (23.9%)	137 (27.5%)	67 (31.6%)	105 (44.7%)		
Parent marital status, *n* (%), *n* = 1288						
Not married	92 (32.7%)	147 (27.6%)	50 (22.1%)	53 (21.4%)	26.6	0.0423
Married	189 (67.3%)	385 (72.4%)	177 (77.9%)	195 (78.6%)		

*Note:* Statistically significant results (*p* < 0.01) are indicated in bold.

Abbreviations: ADHD: attention‐deficit/hyperactivity disorder, PTSD: post‐traumatic stress disorder.

**FIGURE 2:**
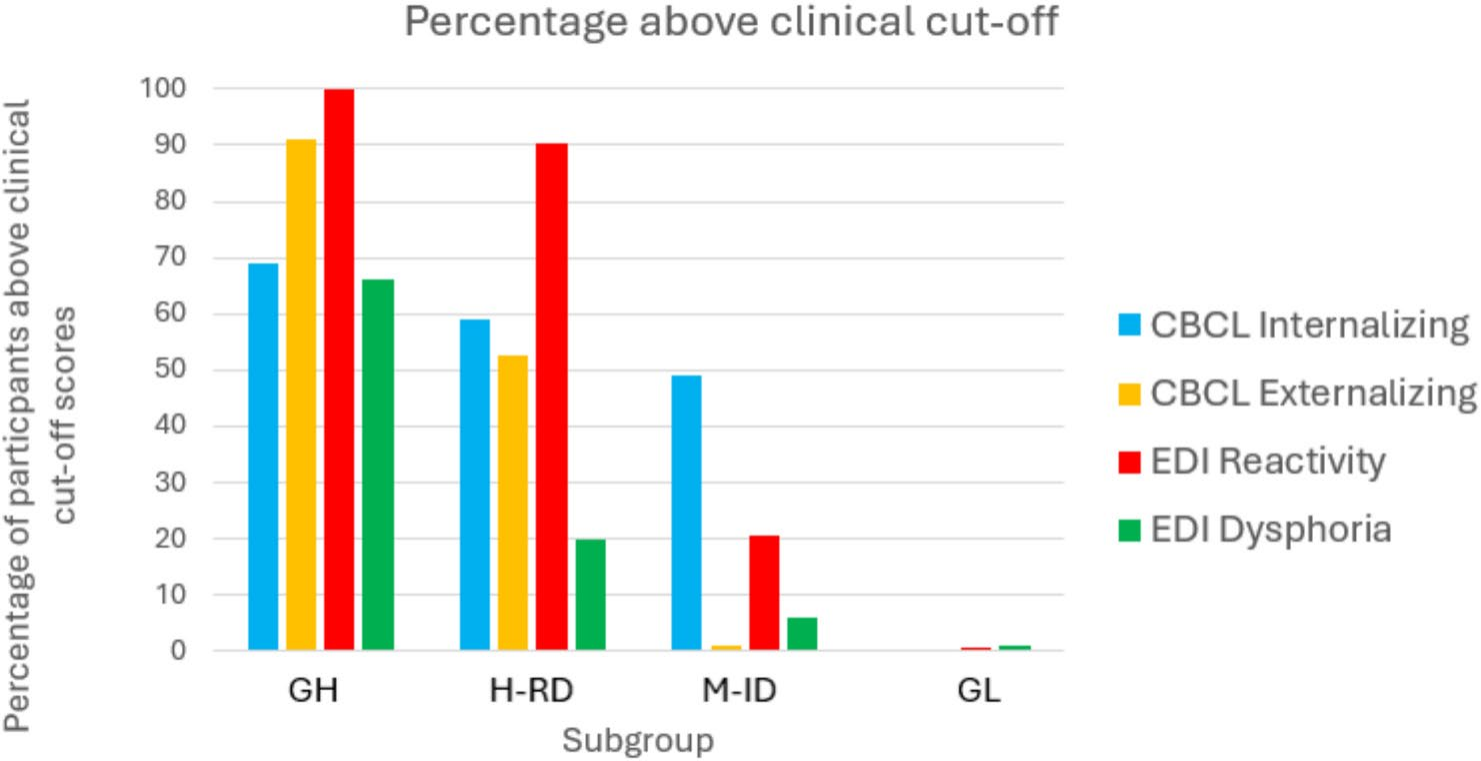
Percentage of autistic children and youth who exhibit scores above clinical cut‐off scores.

Approximately 22% of the sample (*n* = 289) were classified into the GH subgroup, and about 80% of this group came from the inpatient sample. Members of this subgroup demonstrated significantly higher scores compared to all other subgroups. Notably, around 66% had EDI‐Dysphoria scores exceeding the clinical cutoff, while 100% displayed EDI‐Reactivity scores above the clinical range, as can be seen in Figure 2. In terms of externalizing and internalizing behaviors, about 69% of the individuals in this subgroup showed elevated (surpassing the clinical cutoff) CBCL internalizing scores, and approximately 91% had elevated CBCL externalizing scores.

The High—Reactivity Dominant (H‐RD) subgroup comprised 41% of the sample (*n* = 539) and exhibited the second highest levels of subscale scores. Approximately 45% of this subgroup was drawn from the inpatient sample. Notably, around 90% of the individuals in this group had EDI‐Reactivity scores that exceeded the clinical range, while only about 20% surpassed the clinical cutoff for EDI‐Dysphoria. In addition, 59% surpassed the clinical cutoff for CBCL internalizing problems, while approximately 52.5% exceeded the clinical cutoff for CBCL externalizing problems.

Around 18% (*n* = 233) of the sample formed the Moderate–Internalizing Dominant (M‐ID) subgroup, with 26% of this subgroup belonging to the inpatient psychiatric autistic sample. Within this subgroup, approximately 20% have EDI‐Reactivity scores above the clinical cutoff, while only 6% have EDI‐Dysphoria scores above the clinical cutoff range. Half of the sample (49%) was above the cutoff on CBCL internalizing, but only 1% was above the cutoff for CBCL externalizing.

The Global Low (GL) subgroup consisted of 19% (*n* = 250) of the sample, with about 20% of this subgroup belonging to the inpatient psychiatric autism sample. This subgroup exhibits the lowest scores across the four subscales, with only two individuals (0.8%) having EDI‐Reactivity scores above the clinical cutoff, one person (0.4%) scoring above the EDI‐Dysphoria clinical cutoff range, 5 individuals (0.2%) having CBCL‐internalizing scores above the clinical cutoff and none above the CBCL‐externalizing clinical cutoff scores.

### 3.2 Aim 2: Key Clinical Features for Clustering Membership

The feature importance algorithm revealed that individual survey items related to emotional reactivity and aggressive behavior most strongly informed the clustering membership decision. The EDI‐Reactivity items that most strongly informed the clustering decisions were “Has extreme or intense emotional reactions,” “Difficult to distract if upset …,” “Had mood swings,” “Had explosive outbursts,” and “Hard to calm him/her down.” Additionally, the CBCL items “Disobedient at home,” “Stubborn, sullen, or irritable,” “Destroying things belonging to his/her family or others,” “and “Threatens people” were also significant contributors to the clustering decision. On the other hand, individual items related to smoking, alcohol, and drug use showed minimal contribution towards the clustering membership decision.

### 3.3 Aim 3: Sociodemographic Clinical Features and Family Psychiatric History Group Differences

Our analyses showed that the four subgroups did not show any significant differences in child's age, sex assigned at birth, or racial and ethnic identity. Additionally, there were no significant differences among the subgroups in terms of the child's verbal ability, intellectual disability, or severity of autism traits. Subgroup differences were significant for household income (*p* < 0.001) and parental marital status (*p* < 0.05). Additionally, significant differences were observed among the four subgroups based on their family's psychiatric history of bipolar disorder, depression, post‐traumatic stress disorder (PTSD), attention‐deficit/hyperactivity (ADHD), and anxiety (Table 2). Significant results were further examined through post hoc pairwise comparisons to identify specific subgroup pairs that displayed notable differences, utilizing chi‐square tests with Bonferroni correction for multiple comparisons. The subgroups that exhibited the highest number of differences were GH and GL, followed by the pair of subgroups H‐RD and GL. GH and GL subgroups differed significantly on seven demographic and family psychiatric history variables: The GH group had lower household income, *p* < 0.0001, were less likely to report married marital status *p* < 0.001, and were more likely to have a family history of bipolar disorder *p* < 0.0001, PTSD *p* < 0.0001, ADHD *p* < 0.0001, anxiety *p* < 0.01, and depression *p* < 0.0001, as compared to the GL group. The H‐RD and GL subgroups differed significantly on five demographic and family psychiatric history variables: H‐RD had lower household income *p* < 0.0001 and were more likely to have a family history of bipolar disorder *p* < 0.01, PTSD *p* < 0.01, ADHD *p* < 0.0001, and depression *p* < 0.0001, as compared to the GL group.

The results also revealed a significant difference between the GH and M‐ID subgroups in household income *p* < 0.0001, with the GH group having lower household income than the M‐ID group. Between the H‐RD and M‐ID subgroups, H‐RD was significantly more likely to report family history of bipolar disorder, as compared to M‐ID. Finally, for comparisons between the M‐ID and GL subgroups, M‐ID was significantly more likely to report family history of ADHD, *p* < 0.01 and depression *p* < 0.05. Importantly, the GH and H‐RD subgroups showed no significant statistical differences on any of the demographic or family psychiatric history variables.

## 4 Discussion

Numerous researchers have made efforts to identify clinically meaningful autism subtypes. The aim of these endeavors has been to gain insights into the underlying factors associated with autism, with the hope of providing valuable information regarding prognosis and determining appropriate interventions and treatments. The rationale behind this approach is that behaviorally distinct diagnostic subtypes may exhibit specific responses to targeted interventions. To achieve this, researchers have employed cluster analysis, an empirically derived statistical method that utilizes data‐driven techniques to group individuals based on shared characteristics (Hu and Steinberg 2009; Stevens et al. 2017; Matta et al. 2018; Bitsika et al. 2018). Most of this work has focused on core autism characteristics or medical or family features of autism, even though emotional and behavioral challenges often drive treatment seeking, rather than core autism traits (Farley et al. 2009; Joshi et al. 2010).

In this study, cluster analysis was applied to a large, combined inpatient and community sample of autistic youth, resulting in the identification of four distinct subgroups that varied in both severity and type of emotional and behavioral concerns. The results reveal distinct characteristics for each subgroup: in the *Global High Subgroup (GH)*, autistic individuals demonstrate significantly elevated levels of emotion dysregulation, and internalizing and externalizing behaviors, with all participants in this subgroup exceeding clinical cutoffs for at least one of the scales. Demographically, this group exhibits the lowest income levels, the smallest percentage of married parents, and the highest prevalence of family psychiatric histories involving bipolar disorder, depression, and PTSD. This profile of concurrent elevations across all transdiagnostic domains is also consistent with the concept of a general psychopathology “p‐factor,” as proposed within the Hierarchical Taxonomy of Psychopathology (HiTOP) (Kotov et al. 2017). Individuals with a high p‐factor show transdiagnostic elevations across domains—a pattern closely mirroring the GH profile. This framing has important clinical implications: youth in the GH group may benefit most from transdiagnostic treatment approaches targeting shared underlying mechanisms, such as emotion dysregulation, rather than single‐diagnosis‐specific protocols. This is also consistent with the finding that 80% of GH participants came from the inpatient setting, in line with the known association between high p‐factor scores and greater severity of impairment and service utilization.

The *High‐Reactivity Dominant Subgroup (High‐RD)* was the subgroup with the most participants (*n* of 539 as compared to the mid‐200s in each other group); this group shows elevated mean scores in EDI‐Reactivity, with borderline clinical scores in both CBCL Internalizing and Externalizing subscales. Notably, 90% of participants in this group present EDI‐Reactivity scores above the clinical cutoff. This group had the highest percentage of family psychiatric history of anxiety and ADHD. It is important to recognize that the GH and High‐RD groups not only exhibit a higher prevalence of familial mental health disorders but also demonstrate distinct patterns in the specific types of conditions that are most prevalent within each subgroup. Autistic individuals forming the Moderate–Internalizing Dominant Subgroup (M‐ID) display borderline clinical scores on the CBCL Internalizing subscale and mean scores in the typical range for other clinical scales. This subgroup displays the second highest household income, the second largest percentage of married parents, and the second lowest occurrence of family psychiatric history. Finally, the Global Low Subgroup (GL) shows consistently low scores across all subscales, with very few participants exceeding clinical cutoffs on any of the scales. Demographically, the GL subgroup exhibits the highest household income, the greatest percentage of married parents, and the lowest prevalence of family psychiatric histories.

Our results showed that the survey items that most significantly informed the cluster membership decision were primarily items coming from the EDI‐Reactivity and CBCL‐aggression subscales. This finding suggests that the emotional reactivity symptoms and aggressive behaviors captured by these two specific subscales were key distinguishing factors in how the autistic participants were clustered. This observation, alongside previous research in younger samples (Sivathasan et al. 2024), underscores a robust link between reactivity and aggression. Another potential interpretation is that internalizing behaviors may have been less pronounced in this sample, while aggression and reactivity displayed the highest variability across the subgroups. Finally, this finding aligns with primary research suggesting that reactivity may have substantial explanatory power for conceptualization of mental health challenges in autism based on strong associations with a wide range of diagnoses and service utilization (Conner, White, et al. 2020; Conner et al. 2021). Particularly given the prominence of the Reactivity‐Dominant group, these findings overall suggest potential benefits of continuing a research and intervention focus on emotional reactivity in autism.

The post hoc analyses showed distinct patterns in household income and parental marital status among the four subgroups. Moreover, the GL subgroup (lowest emotional/behavioral concerns) had the highest proportion of non‐ or minimally verbal participants, whereas the GH group had the lowest. This suggests that, contrary to assumptions, verbal ability may not necessarily be linked to more compromised emotional and behavioral symptoms. This result is consistent with other research that has found more fluent verbal ability to be associated with higher levels of EDI‐Reactivity (Northrup et al. 2024, 2021). It is possible that children with lower verbal abilities tend to be placed in more accommodating or structured environments that help to reduce emotional and behavioral challenges. Moreover, it is important to highlight that we used an approximate measure of speaking ability (e.g., ADOS‐2 module, parent report). There are other forms of communication that were not measured as part of this study that children might employ. Nonverbal communication, such as gestures, eye contact, or even play, can provide significant insights into a child's emotional state and needs and require further future study.

The data suggested that autistic youth from the most resource‐constrained and psychologically challenged households—characterized by low incomes, single parenthood, and elevated family histories of mental health conditions—are the most likely to experience the greatest difficulties in regulating their emotions and behaviors. Conversely, those from more affluent, stable family environments with fewer mental health concerns appear to exhibit the most emotional and behavioral well‐being. These intersecting sociodemographic and psychiatric history variables underscore the need for a nuanced, holistic understanding of the lived experiences of autistic individuals and their families to develop tailored, equity‐focused supports.

This aligns with existing literature on the impact of socioeconomic status on the mental health of autistic individuals (Liao and Li 2020; Weiss et al. 2021; Karst and van Hecke 2012; Vasilopoulou and Nisbet 2016). Families with lower incomes often experience significant financial stress due to the costs of therapies and special education, exacerbating mental health challenges for both parents and children (Karst and van Hecke 2012; Weiss et al. 2021; Liao and Li 2020). Parents of autistic children frequently experience higher stress levels, often leading to anxiety and depression, which can negatively affect their children's well‐being (Karst and van Hecke 2012). In contrast, higher‐income families generally have better access to support systems that can alleviate stress and promote healthier environments for autistic children.

Previous research has established that emotional and behavioral issues in autistic individuals are more closely linked to family functioning (McStay et al. 2014; Pozo et al. 2014) and parental well‐being (Vasilopoulou and Nisbet 2016) than to the severity of core autism characteristics. Our findings additionally highlight the role of family mental health history in shaping the outcomes of autistic children and youth. Beyond psychiatric history, several broader family environment factors not assessed in the present study warrant consideration in future research. Family‐level dynamics—including parent–child interaction quality, interparental conflict, and sibling relationships—have each been linked to emotional and behavioral challenges in autistic individuals (Woodman et al. 2016; MacKenzie et al. 2025; Northrup et al. 2024). School environment also plays a role, as academic stress, inadequate support, and difficult peer relationships may exacerbate difficulties, particularly for verbally fluent youth facing higher academic demands (Humphrey and Hebron 2015). Moreover, our findings call for a more in‐depth investigation into potential genetic contributions that may be associated with these emotional and behavioral challenges in autistic individuals (Piven et al. 1997). These findings, when considered alongside the previous insights about socioeconomic status and family structure, paint a complex picture of the heterogeneity observed in autistic youths' emotional and behavioral presentations.

Ultimately, results of the current study underscore the importance of carefully considering an autistic youth's scores on the EDI‐Reactivity and CBCL‐aggression subscales when developing targeted interventions. For youth in the GH and H‐RD subgroups—characterized by high emotional reactivity—clinicians may wish to prioritize emotion dysregulation as a primary treatment target. Several interventions have been developed and tailored to target emotion regulation in autistic people, across various ages and levels of caregiver involvement, including young children (The Stress and Anger Management Program [STAMP]), school‐age children and adolescents (Regulating Together), and adolescents and adults (the Emotion Awareness and Skills Enhancement [EASE] program and Dialectical Behavior Therapy [DBT]) (Factor et al. 2019; Shaffer et al. 2023; Conner et al. 2019; Beck et al. 2022; Hartmann et al. 2019). Notably, most existing interventions have been developed and tested with verbally fluent, intellectually able individuals; interventions specifically targeting emotion regulation in minimally speaking autistic youth or those with intellectual disability remain critically lacking and represent an important gap for future research. Targeting emotion regulation is theorized to yield downstream reductions in aggressive and other safety‐compromising behaviors, though more work is needed to establish these effects as secondary behavioral targets. As aggressive behavior was a secondary driver of membership in these groups, it will be important for clinicians to consider targeting aggression through evidence‐based interventions such as PCIT‐Autism (Allen et al. 2023) and the RUBI parent training program (Bearss et al. 2015), which offer evidence‐based strategies for behavioral management in autistic youth; more work is needed to target aggression in autistic adolescents and adults, with emerging work focused on CBT for aggression (Sukhodolsky et al. 2016). For youth in the M‐ID subgroup, whose profile is characterized primarily by internalizing problems, modified cognitive behavioral therapy and behavioral activation approaches may be most appropriate (Menezes et al. 2024; Bal et al. 2025; Wood et al. 2015; Schwartzman et al. 2024). For the GL subgroup, universal prevention and family support approaches may suffice. Of note, much of the intervention research to date has employed highly trained clinicians who are specialized in working with autistic clients and their families, and thus it is unclear whether these interventions would provide similar outcomes with less trained providers (Maddox et al. 2021). Moreover, while reactivity and aggression were the main factors influencing subgroup membership decisions, different patterns regarding internalizing problems and dysphoria emerged among the four identified subgroups, which may suggest important individual‐level intervention targets and skills to focus on. Importantly, future research is needed to determine if our empirically derived subgroups do require different approaches (psychosocial or medication) or respond differentially to different interventions.

## 5 Limitations

Study methods need to be considered when interpreting the findings. All data was derived from caregiver reports, which offer valuable insights but may reflect subjective perspectives. The reliance on caregiver‐report measures, while ecologically valid, may not fully capture the autistic individual's own experience of emotional and behavioral challenges; future research should incorporate self‐report and multi‐informant approaches. Incomplete data was excluded to preserve the integrity of the clustering analyses, as missing values can bias distance calculations and distort cluster structures. Data imputation and sensitivity analyses can be found in the Supporting Information S2. However, this approach may have reduced the representativeness of the overall sample. To support interpretability, we chose items from the CBCL internalizing and externalizing scales as well as both EDI scales. We felt this captured a broad range of items, capturing many of the most common concerns. Nonetheless, there are other aspects of mental health (i.e., attention, psychotic symptoms, etc.) not captured that warrant future study. Furthermore, the discrepancies in measurement methods for intellectual disability and verbal ability across both datasets restrict the depth of analysis for these variables. Additionally, we did not have comprehensive medical and psychiatric histories for the youth participants, which could have provided valuable context for interpreting behaviors and outcomes. The CBCL was normed on a general population sample, and T‐score cutoffs may not perfectly translate to autistic youth; some items may have elevated base rates in this population for reasons unrelated to psychopathology. Although our sample spans the full clinical severity range from community to inpatient settings, it is demographically homogeneous (85.7% white, 92% non‐Hispanic/non‐Latino, 73% married parents, over 40% with household income above $81,000) and therefore does not reflect the full diversity of the autistic population. These demographic characteristics limit the generalizability of our findings, particularly for autistic youth from racially and ethnically minoritized communities and from lower‐income households and underscore the need for future research with more diverse samples.

## 6 Conclusions

By utilizing cluster analysis, researchers can uncover meaningful subgroups within the autistic population that demonstrate unique emotional and behavioral profiles. The identified subgroups vary substantially, including those with global patterns of high or very low/no problems across domains as well as subgroups characterized by reactive or internalizing presentations. These subgroups would most certainly require different forms of support and intervention. Our identification of these subgroups is a first step that can direct future work that incorporates other data on possible risk and protective or differentiating factors between groups. Our findings also show that autistic youth from resource‐limited households—characterized by low income, single‐parent structures, and significant family mental health challenges—face the greatest difficulties in emotional and behavioral regulation. The interplay of these sociodemographic and psychiatric factors underscores the need for a nuanced understanding of the experiences of autistic individuals and their families to develop customized, equity‐based support.

## Author Contributions

**Safaa Eldeeb:** conceptualization, formal analysis, writing – original draft. **Jessie Northrup**, **Caitlin Conner**, **Shalini Sivathasan**, and **Ligia Antezana:** review and editing. **Matthew Siegel:** data collection, resources, review and editing. **Carla Mazefsky:** conceptualization, funding acquisition, supervision, writing – review and editing.

## Funding

This research was funded by the National Institutes of Health (NIH) grant R01HD079512 (to CM) with support from UL1TR001857. During preparation of this manuscript, Drs. Eldeeb and Antezana were supported by the National Institute of Mental Health (NIMH) T32 MH018951, and Dr. Northrup was supported by NIMH K23MH127420.

Data collection was supported with funding from NICHD R01 HD079512 (CM), Simons Foundation Autism Research Initiative (SFARI #296318, M.S.) and the Nancy Lurie Marks Family Foundation. IAN is a partnership project of the Kennedy Krieger Institute and the Simons Foundation. IAN was also partially funded through a Patient‐Centered Outcomes Research Institute (PCORI) Award for development of the National Patient‐Centered Clinical Research Network, known as PCORnet.

We extend our sincere gratitude to the participants and their caregivers for their time, commitment, and contributions.

## Ethics Statement

This study received ethical approval from the following Institutional Review Boards (IRBs):

- University of Maryland: IRB Protocol #536200 (approved 2014).
- Maine: IRB Protocol #958815 (approved December 20, 2013).
- Cincinnati: IRB Protocol #2014–1750 (approved May 7, 2014).
- Colorado: IRB Protocol #14–0035 (approved March 7, 2014)
- Rhode Island: IRB Protocol #4004–16 (approved February 13, 2014).
- University of Pittsburgh (AIC STUDY): Protocol #19050171.
- IAN: CR00055351.

The study (ADDIRC) was conducted across four phases with a total enrollment of 1954 participants:

- Phase 1: February 2014—September 2015 (493 enrolled).
- Phase 2: October 2015—November 2018 (917 enrolled).
- Phase 3: December 2018–September 2022 (471 enrolled, including 65 double phases).
- Phase 4: October 2022—November 2024 (139 enrolled).

## Consent

Consent was obtained from all adult participants. For minor participants, parental/guardian consent and child assent were obtained as appropriate.

## Conflicts of Interest

The authors declare no conflicts of interest.

## Supporting information


**Table S2:1** CBCL item‐level missingness distribution (among IAN participants with complete EDI data).
**Table S2:2** Data missingness AIC inpatient sample.
**Table S2:3** Missing data mechanism test (IAN).
**Table S2:4** Multiple Imputation Sensitivity Analysis (IAN).
**Table S2:5** Multiple Imputation Sensitivity Analysis (AIC).
**Table S2:6** AIC Per‐Imputation ARI and Subgroup Proportions.

## Data Availability

The data that support the findings of this study are available from the corresponding author upon reasonable request.
