# Supplementary material for: Identifying Unique Subgroups of Emotional and Behavioral Presentations in a Large Inpatient and Community Sample of Autistic Youth
Source: Autism Res. 2026 Jun 26;19(8):e70307. doi: 10.1002/aur.70307 (PMC13471816; doi:10.1002/aur.70307)
Supplement: Supplementary file 1 [file AUR-19-0-s001.docx]

**Supplemental S1: Detailed Description of Study Measures and Instruments.**

**Sociodemographic variables.** Caregivers were asked to report on caregiver and child sociodemographic characteristics, including their child’s age, sex assigned at birth, and racial and ethnic identity. Caregivers also reported their household income, marital status and their immediate family's history of bipolar disorder, depression, anxiety, attention deficit hyperactivity disorder (ADHD), and post-traumatic stress disorder (PTSD).

**Clinical Features**

Verbal ability was determined by the ADOS-2 for the inpatient participants and by parent report for the community participants. The inpatient sample participants were considered fluently speaking if that received the ADOS-2 module 3 or 4, which requires fluent, meaningful speech. The community participants were considered fluently speaking if they were rated as having “meaningful, fluent speech” in response to the question “how would you best describe (child name's) current verbal ability”. Intellectual ability was evaluated using a direct assessment of the nonverbal intelligence quotient (NVIQ) standard score from the Leiter International Performance Scale-Third Edition in the inpatient sample. In the community sample, caregivers answered the question, “What was (child’s name) most recent IQ score?” with response options provided in ranges. Autism-related characteristics were assessed using the Social Communication Questionnaire (SCQ). The SCQ-Lifetime version^1^ is a 40-item questionnaire assessing current and lifetime autistic traits. Each dichotomous item is scored based on the presence or absence of a behavior.

**Emotion Dysregulation Inventory.** The Emotion Dysregulation Inventory (EDI) is a proxy-report measure characterizing the severity of dysregulation across two dimensions: Reactivity (fast, intense emotional reactions) and dysphoria (low positive affect, sadness, unease) ^2^. The reactivity subscale consists of 24 items, while the dysphoria subscale consists of 6 items. Items are rated on a five-point scale of problem severity over the past 7 days ranging from (Not at all) to (Very Severe). The EDI was developed and validated for autistic children and adolescents. Clinical cutoffs were established in the original validation study ^2,3^using item response theory calibration with normative comparisons drawn from a general U.S. population sample (N = 1,380). ). Clinical cutoffs are defined as T-scores at or above 60 for both the Reactivity and Dysphoria subscales, corresponding to one standard deviation or more above the general population mean - the threshold conventionally used to identify clinically elevated scores. Exceeding this cutoff on the Reactivity subscale indicates that the individual’s emotional reactivity is substantially more severe than that of the typical person in the general population and is associated with higher rates of psychiatric hospitalization and greater service utilization^4^.

**Child Behavior Checklist.** The Child Behavior Checklist (CBCL) is a widely utilized caregiver-report measure for assessing psychiatric symptoms ^5^. It involves scoring items based on the past month using a scale of 0 (not true), 1 (occurs sometimes), and 2 (occurs often). In this study, we utilized survey items from both the internalizing and externalizing subscales. The internalizing subscale encompasses items related to anxious/depressed, withdrawn/depressed, and somatic complaints, while the externalizing subscale includes items from the attention problems, rule-breaking, and aggressive behavior subscales. It should be noted that the CBCL was normed on a general population sample, and T-score interpretations therefore assume a neurotypical reference group. Some items (e.g., somatic complaints, social withdrawal) may have elevated base rates in autistic youth for reasons unrelated to psychopathology, and caregiver distress may influence ratings. Because we used item-level CBCL data as input to the clustering algorithm rather than T-scores, reliance on norm-referenced interpretations is reduced, though not eliminated. The T-scores reported in Table 2 are used descriptively to characterize subgroups, and clinical cutoffs are interpreted with this caveat in mind. We also note that the CBCL has nonetheless demonstrated at least preliminary psychometric support for use with autistic populations (see, e.g.,(Pandolfi et al., 2012)), and it has been widely employed across autism research, providing a basis for meaningful cross-study comparison.

**Conceptual distinction between the EDI-Reactivity and CBCL-Aggression subscales.** We want to clarify a key aspect of our analytic approach that the clustering analysis was conducted entirely at the item level. Individual survey items from both the EDI and CBCL were the inputs to the random forests ensemble clustering algorithm - not subscale scores. Because random forests select random subsets of features at each split, the algorithm is robust to multicollinearity among individual items; overlapping items do not inflate or distort the clustering solution. Item-level overlap across what we subsequently label "subscales" therefore has no methodological impact on the primary analysis or its results. Subscale scores (EDI-Reactivity, EDI-Dysphoria, CBCL Internalizing, and CBCL Externalizing) were used only descriptively, after clustering, to characterize and interpret the four subgroups in a clinically meaningful way.

At the interpretive level, the EDI and CBCL do assess overlapping but meaningfully distinct constructs. The EDI was developed and validated specifically for autistic individuals (Mazefsky et al., 2018) and frames dysregulation as an internal emotional process - the speed, intensity, and duration of negative emotional arousal and difficulty modulating that arousal. The CBCL Aggression subscale, by contrast, is behaviorally anchored, indexing observable externalizing acts without reference to the underlying emotional process. To quantify empirical overlap, we computed bivariate correlations between EDI-Reactivity total scores and CBCL Aggression subscale scores within each sample: IAN community sample, r = .72 (p < .001; shared variance ~52%); AIC inpatient sample, r = .49 (p < .001; shared variance ~24%). These confirm the scales are substantially related - consistent with the known co-occurrence of emotional reactivity and aggression in autistic youth - while retaining 48-76% unique variance, supporting their interpretive distinctiveness.

**Supplemental S2: Missing Data Patterns and Multiple Imputation Sensitivity Analysis**

*Identifying Unique Subgroups with Varied Emotional and Behavioral Presentations in a Large, Combined Inpatient and Community Sample of Autistic Youth*

**Overview**

This supplement reports: (1) patterns and proportions of missing data in both study samples; (2) results of a formal test of the missing data mechanism in the IAN sample; and (3) a multiple imputation sensitivity analysis evaluating the stability of the primary complete-case clustering solution.

**Part 1. Missing data patterns**

***IAN community sample***

**Total eligible:** 1,176 age-eligible IAN participants (ages 6-17).

**EDI missingness:** Of the 1,176 age-eligible IAN participants, 64 had incomplete EDI item-level data and were excluded prior to CBCL assessment. Among those who submitted an EDI form, item-level missingness was minimal; exclusions were driven by participants who did not complete the form at all rather than by sporadic missing items.

**CBCL missingness:** Missing CBCL data was the primary driver of exclusion in the IAN sample. Table S2.1 below summarizes the distribution of CBCL item-level missingness among the 1,112 participants who had complete EDI data.

**Analysis sample:** 865 participants with complete item-level data on both the EDI and CBCL were retained for the primary analysis.

**Analysis sample:** 865 participants with complete item-level data on both the EDI and CBCL were retained for the primary analysis.

Table S2.1. CBCL item-level missingness distribution (among IAN participants with complete EDI data):

| **Missing CBCL items per participant** | **N participants** | **Notes** |
| --- | --- | --- |
| 0 (fully complete) | 865 | Included in analysis |
| 1 item missing | 241 | Recoverable with imputation |
| 2 items missing | 56 | Recoverable with imputation |
| 3–16 items missing | 47 | Borderline |
| All 119 missing (no form) | 111 | Structural - no CBCL collected |

***AIC inpatient sample***

**Total eligible:** 1,356 AIC participants; 1,242 were ages 6–17.

**EDI missingness:** Of the 1,242 age-eligible AIC participants, 401 were excluded due to no or partial EDI data. These exclusions were predominantly Phase 1 participants for whom the EDI had not yet been introduced, rather than participants who declined or partially completed the form.

**Primary source of missingness:** Structural. The CBCL was not administered during Phase 1 of data collection. Consequently, 284 of the 1,242 age-eligible participants had no EDI data (primarily Phase 1 participants), and 559 had no CBCL data. Among participants with at least some data on both instruments, 446 had complete item-level data on both the EDI and CBCL.

**CBCL missingness:** Of the 841 participants with complete EDI data, a further 395 were excluded due to missing one or more CBCL items, again primarily reflecting Phase 1 and early Phase 2 participants for whom the CBCL was not yet part of the data collection protocol.

**Analysis sample:** 446 AIC participants with complete item-level data on both the EDI and CBCL were retained for the primary analysis. Table S2.2 summarizes these exclusions sequentially.

Table S2.2. Data missingness AIC inpatient sample.

| **Filter stage** | **N remaining** | **N dropped** | **Reason** |
| --- | --- | --- | --- |
| Total AIC | 1,356 | - | - |
| Age 6–17 | 1,242 | 114 | Outside age range |
| + EDI complete | 841 | 395 | No/partial EDI (Phase 1) |
| + CBCL complete | 417 | 424 | Missing ≥1 CBCL item |
| 0 (fully complete) | 446 | - | Included in analysis |

**Part 2. Missing data mechanism test (IAN)**

To assess whether missing data in the IAN sample were related to key participant characteristics, we compared included versus excluded participants on age, sex assigned at birth, and race using independent-samples t-tests and chi-square tests.

Table S2.3. Missing data mechanism test (IAN)

| **Variable** | **Included** | **Excluded** | **Statistic** | **p-value** |
| --- | --- | --- | --- | --- |
| Age, mean (SD) | 11.94 (3.17) | 12.42 (3.27) | t = −2.60 | .009 |
| % Male | 81.8% | 73.8% | χ² = 11.24 | .001 |
| % White | 92.1% | 90.2% | χ² = 1.24 | .266 |

*Note.* Excluded participants were slightly older and less likely to be male than included participants; racial composition did not differ significantly. These differences are modest in magnitude and are unlikely to substantially bias the primary cluster solution.

For the AIC sample, missingness was predominantly structural: the CBCL was not administered during Phase 1 of data collection, meaning the majority of excluded participants lacked data due to study design rather than participant non-response or dropout. A formal MAR test comparing included versus excluded AIC participants was therefore not conducted, as the missingness mechanism is known a priori and is independent of participant characteristics. Among the subset of AIC participants for whom both instruments were introduced (Phases 2–3), item-level missingness was minimal, and the 446 participants with fully complete data did not differ systematically from those with partial data in terms of study design eligibility criteria.

**Part 3. Multiple imputation sensitivity analysis (IAN)**

***Method***

To evaluate whether the complete-case findings were sensitive to missing data, we conducted a sensitivity analysis using multiple imputation by chained equations^6,7^. Twenty imputed datasets were generated for the IAN sample using predictive mean matching, with the imputation pool restricted to participants who had a completed CBCL form and complete EDI data (N = 1,159), thus excluding the 111 participants with no CBCL form completed.

The four-cluster solution was re-applied to each imputed dataset using the same clustering approach as the primary analysis. Agreement between each imputed-data cluster solution and the complete-case solution was quantified using the Adjusted Rand Index (ARI;^8,9^). The ARI ranges from 0 (chance agreement) to 1 (perfect agreement). Cluster label matching across imputations was performed using the Hungarian algorithm to ensure proportions are comparable across imputed datasets.

***Results***

**Cluster stability.** Across all 20 imputed datasets, the mean ARI comparing each imputed solution to the complete-case solution was 0.84 (SD = 0.06, range 0.78–0.94). The four-subgroup structure was reproduced in every imputed dataset without exception.

**Subgroup proportions.** Subgroup proportions in the imputed solutions closely mirrored those of the complete-case analysis, as shown in Supplemental Table S2.4.

Table S2.4. **Multiple Imputation Sensitivity Analysis (IAN)**

| **Subgroup** | **Complete-case %** | **Imputed mean %** | **Imputed range** |
| --- | --- | --- | --- |
| Global High (GH) | 22% | 12% | 11–13% |
| High-Reactivity Dominant (H-RD) | 41% | 19% | 17–21% |
| Moderate-Internalizing Dominant (M-ID) | 18% | 27% | 26–29% |
| Global Low (GL) | 19% | 42% | 40–44% |

*Note.* Complete-case proportions reflect the full combined sample (IAN + AIC; N = 1,311). Imputed proportions reflect the IAN-only imputation pool (N = 1,159). The proportion differences reflect the different sample composition rather than instability in cluster structure. ARI = 0.84 (range 0.78–0.94) confirms high agreement between imputed and complete-case solutions on the IAN sample alone.

**Part 4. Multiple Imputation Sensitivity Analysis (AIC)**

**Method.** To evaluate whether the AIC complete-case findings were sensitive to the stochastic (non-structural) component of missing data, we conducted a parallel MICE sensitivity analysis for the AIC sample. Twenty imputed datasets were generated using predictive mean matching (scikit-learn IterativeImputer, max_iter = 10), with the imputation pool restricted to participants with at least partial item-level data on both the EDI and CBCL. This excluded participants for whom one or both instruments were entirely absent (structural missingness), consistent with the recommendation that imputation cannot recover data that were never collected.

The four-cluster solution was re-applied to each imputed dataset using identical parameters to the primary analysis. Agreement with the complete-case solution was quantified using the ARI, with Hungarian algorithm label alignment across imputations.

**Results. Cluster stability.** Across all 20 imputed datasets, the mean ARI was 0.886 (SD = 0.011, range = 0.883–0.932), indicating very high agreement between each imputed-data solution and the complete-case solution. The four-subgroup structure was reproduced in all 20 imputed datasets without exception.

**Subgroup proportions.** Subgroup proportions across imputed solutions were highly stable, as shown in Table S2.5 below.

Table S2.5. **Multiple Imputation Sensitivity Analysis (AIC)**

| **Subgroup** | **Complete-case %** | **Imputed mean %** | **Imputed range** |
| --- | --- | --- | --- |
| Global High (GH) | 23.6% | 23.2% | 23.1–24.4% |
| High-Reactivity Dominant (H-RD) | 38.8% | 36.2% | 35.7–36.4% |
| Moderate-Internalizing Dominant (M-ID) | 14.7% | 19.3% | 18.4–19.4% |
| Global Low (GL) | 22.8% | 21.3% | 21.2–21.5% |

*Note. Complete-case proportions reflect AIC complete-case sample (N = 446). Imputed proportions reflect the AIC imputation pool. Subgroup labels were aligned to the complete-case solution using the Hungarian algorithm. ARI = 0.886 (SD = 0.011, range 0.883–0.932), confirming high agreement between imputed and complete-case solutions. The proportion differences between complete-case and imputed solutions are modest and do not suggest instability in cluster structure.*

**Table S2.6. AIC Per-Imputation ARI and Subgroup Proportions**

| **Imputation** | **ARI** | **GH %** | **H-RD %** | **M_ID %** | **GL %** |
| --- | --- | --- | --- | --- | --- |
| 1 | 0.8828 | 23.1% | 36.4% | 19.4% | 21.2% |
| 2 | 0.8908 | 23.2% | 36.2% | 19.0% | 21.5% |
| 3 | 0.8908 | 23.2% | 36.2% | 19.0% | 21.5% |
| 4 | 0.8828 | 23.2% | 36.2% | 19.4% | 21.2% |
| 5 | 0.8828 | 23.2% | 36.2% | 19.4% | 21.2% |
| 6 | 0.8828 | 23.2% | 36.2% | 19.4% | 21.2% |
| 7 | 0.8828 | 23.2% | 36.2% | 19.4% | 21.2% |
| 8 | 0.8874 | 23.1% | 36.2% | 19.4% | 21.4% |
| 9 | 0.8828 | 23.2% | 36.2% | 19.4% | 21.2% |
| 10 | 0.8828 | 23.2% | 36.2% | 19.4% | 21.2% |
| 11 | 0.8828 | 23.2% | 36.2% | 19.4% | 21.2% |
| 12 | 0.8828 | 23.2% | 36.2% | 19.4% | 21.2% |
| 13 | 0.8828 | 23.2% | 36.2% | 19.4% | 21.2% |
| 14 | 0.8828 | 23.2% | 36.2% | 19.4% | 21.2% |
| 15 | 0.9318 | 24.4% | 35.7% | 18.4% | 21.5% |
| 16 | 0.8828 | 23.2% | 36.2% | 19.4% | 21.2% |
| 17 | 0.8828 | 23.2% | 36.2% | 19.4% | 21.2% |
| 18 | 0.8828 | 23.2% | 36.2% | 19.4% | 21.2% |
| 19 | 0.8828 | 23.2% | 36.2% | 19.4% | 21.2% |
| 20 | 0.8828 | 23.2% | 36.2% | 19.4% | 21.2% |

*Note. ARI = Adjusted Rand Index (0 = chance agreement, 1 = perfect agreement). Cluster proportions are of the imputation pool (N = 594). Cluster labels aligned via Hungarian algorithm.*

**Conclusion**

The high mean ARI and consistent four-subgroup structure across all 20 imputed datasets provide strong evidence that the primary complete-case findings are robust to the observed pattern of missing data in both samples. For the IAN sample, the mean ARI of 0.84 (range 0.78–0.94) confirms that the cluster solution is stable under imputation of stochastic missingness. For the AIC sample, the mean ARI of 0.886 (range 0.883–0.932) - slightly higher than IAN, reflecting the smaller proportion of imputed participants in the pool - likewise confirms robustness. Structural missingness (Phase 1 AIC participants for whom instruments were not administered) was excluded from imputation in both analyses, consistent with standard recommendations. The complete-case findings for both samples are therefore presented as primary results, with this supplemental analysis confirming their robustness.

***References***

1.	Rutter M, Bailey A, Lord C. *The Social Communication Questionnaire*. Springer New York; 2003. doi:10.1007/978-1-4419-1698-3

2.	Mazefsky CA, Yu L, White SW, Siegel M, Pilkonis PA. The emotion dysregulation inventory: Psychometric properties and item response theory calibration in an autism spectrum disorder sample. *Autism Research*. 2018;11(6):928-941. doi:10.1002/aur.1947

3.	Mazefsky CA, Day TN, Siegel M, White SW, Yu L, Pilkonis PA. Development of the Emotion Dysregulation Inventory: A PROMIS®ing Method for Creating Sensitive and Unbiased Questionnaires for Autism Spectrum Disorder. *J Autism Dev Disord*. 2018;48(11). doi:10.1007/s10803-016-2907-1

4.	Conner CM, Golt J, Shaffer R, Righi G, Siegel M, Mazefsky CA. Emotion Dysregulation is Substantially Elevated in Autism Compared to the General Population: Impact on Psychiatric Services. *Autism Research*. 2021;14(1):169-181. doi:10.1002/aur.2450

5.	Achenbach T, Rescorla LA. Manual for the ASEBA School-age Forms & Profiles: An Integrated System of Multi-informant Assessment. . *University of Vermont, Research Center for Children, Youth & Families*. Published online 2001.

6.	van Buuren S, Groothuis-Oudshoorn K. mice: Multivariate imputation by chained equations in R. *J Stat Softw*. 2011;45(3). doi:10.18637/jss.v045.i03

7.	Mazza GL, Enders CK, Ruehlman LS. Addressing Item-Level Missing Data: A Comparison of Proration and Full Information Maximum Likelihood Estimation. *Multivariate Behav Res*. 2015;50(5). doi:10.1080/00273171.2015.1068157

8.	Osamor IP, Osamor VC. OsamorSoft: clustering index for comparison and quality validation in high throughput dataset. *J Big Data*. 2020;7(1). doi:10.1186/s40537-020-00325-6

9.	Steinley D. Properties of the Hubert-Arabie adjusted Rand index. *Psychol Methods*. 2004;9(3). doi:10.1037/1082-989X.9.3.386
